# *PacCYP707A2* negatively regulates cherry fruit ripening while *PacCYP707A1* mediates drought tolerance

**DOI:** 10.1093/jxb/erv169

**Published:** 2015-05-08

**Authors:** Qian Li, Pei Chen, Shengjie Dai, Yufei Sun, Bing Yuan, Wenbin Kai, Yuelin Pei, Suihuan He, Bin Liang, Yushu Zhang, Ping Leng

**Affiliations:** ^1^College of Agronomy and Biotechnology, China Agricultural University, Beijing 100193, PR China; ^2^Department of Chemistry and Biochemistry, University of Arizona, 1306 East University BouleVard, Tucson, AZ, USA

**Keywords:** ABA, *CYP707A*, cherry ripening, colouring, dehydration stress, particle bombardment, VIGS.

## Abstract

Sweet cherry is a non-climacteric fruit and its ripening is regulated by abscisic acid (ABA) during fruit development. In this study, four cDNAs (*PacCYP707A1*–*4*) encoding 8′-hydroxylase, a key enzyme in the oxidative catabolism of ABA, were identified in sweet cherry fruits using tobacco rattle virus-induced gene silencing (VIGS) and particle bombardment approaches. Quantitative real-time PCR confirmed significant down-regulation of target gene transcripts in VIGS-treated cherry fruits. In *PacCYP707A2*-RNAi-treated fruits, ripening and fruit colouring were promoted relative to control fruits, and both ABA accumulation and *PacNCED1* transcript levels were up-regulated by 140%. Silencing of *PacCYP707A2* by VIGS significantly altered the transcripts of both ABA-responsive and ripening-related genes, including the ABA metabolism-associated genes *NCED* and *CYP707A*, the anthocyanin synthesis genes *PacCHS, PacCHI, PacF3H, PacDFR, PacANS*, and *PacUFGT*, the ethylene biosynthesis gene *PacACO1*, and the transcription factor *PacMYBA*. The promoter of *PacMYBA* responded more strongly to *PacCYP707A2*-RNAi-treated fruits than to *PacCYP707A1*-RNAi-treated fruits. By contrast, silencing of *PacCYP707A1* stimulated a slight increase in fruit colouring and enhanced resistance to dehydration stress compared with control fruits. These results suggest that *PacCYP707A2* is a key regulator of ABA catabolism that functions as a negative regulator of fruit ripening, while *PacCYP707A1* regulates ABA content in response to dehydration during fruit development.

## Highlight

*PacCYP707A2* plays a primary role in regulating ABA levels during the onset of cherry fruit ripening, while *PacCYP707A1* regulates the ABA content in response to dehydration.

## Introduction

The fruits of the Sweet Cherry tree are classified as non-climacteric (Ishiguro *et al.*, 1993). Cherry fruits exhibit a biphasic growth pattern in which initiation of ripening is coupled with ABA increasing (Ren *et al.*, 2010; Luo *et al.*, 2014). By comparison with other deciduous tree fruits, cherry development is rapid; only 50 d are needed for complete development from pollination to full ripening. Thus, cherry represents a potentially valuable model for the study of fruit maturation and ripening (Yoo *et al.*, 2003; María and Basanta *et al.*, 2014). As a typical non-climacteric fruit, ethylene production is very low during the ripening process (Gong *et al.*, 2002; Luo *et al.*, 2014) and ripening is mainly regulated by ABA (Kondo and Inoue, 1997; Setha *et al.*, 2005; Soto *et al.*, 2013). Endogenous ABA levels are regulated via a dynamic balance between biosynthesis mediated by *PacNCED*s, and catabolism mediated by *PacCYP707A*s, with the transcriptional regulation of these genes influencing the ripening process (Kondo and Gemma, 1993; Ren *et al.*, 2010). Changes in the dynamic balance of these two processes may determine ABA levels. In higher plants the ABA biosynthetic pathway is well understood, and ABA is known to be synthesized *de novo* from a C_40_ carotenoid. An important phase of ABA biosynthesis is initiated in plastids with the hydroxylation and epoxidation of β-carotene to produce the all-*trans*-xanthophylls zeaxanthin and violaxanthin. Violaxanthin is then converted to 9-*cis*-epoxyxanthophylls, which are oxidatively cleaved by 9-*cis*-epoxycarotenoid dioxygenases (NCEDs) to yield xanthoxin, the first C_15_ intermediate of ABA biosynthesis. Xanthoxin exits the plastid and is oxidized in the cytosol in two further steps to generate ABA (Qin and Zeevaart, 1999; Schwartz *et al.*, 2003; Taylor *et al.*, 2005). ABA catabolism proceeds predominantly via hydroxylation of the C-8′ methyl group, which is mediated in *Arabidopsis* by a cytochrome P450 (CYP) monooxygenase encoded by a member of the *CYP707A* gene family (Krochko *et al.*,1998; Saito *et al.*, 2004; Kushiro *et al.*, 2004). CYP707A, the key ABA catabolic enzyme, was first characterized in *Arabidopsis* (Kushiro *et al.*, 2004; Saito *et al.*, 2004), and has since been cloned and characterized from various climacteric fruit species including tomato (Sun *et al.*, 2012*a*, *b*) and persimmon (Zhao *et al.*, 2012), as well as non-climacteric fruits such as pear (Dai *et al.*, 2014) and grape (Sun *et al.*, 2010). *CYP707A* genes are involved in several physiological processes including seed dormancy and germination, dehydration and rehydration, and stomatal movement (Millar *et al.*, 2006; Okamoto *et al.*, 2006). In recent years, considerable progress has been made in our understanding of ABA signal transduction (Ma *et al.*, 2009; Melcher *et al.*, 2009; Nishimura *et al.*, 2009; Park *et al.*, 2009), and epigenetic mechanisms for ABA signalling have been reported (Zhang *et al.*, 2011, 2012; Ding *et al.*, 2014). Furthermore, it is reported that ABA might directly regulate the expression of *PacMYBA*, a transcription factor that interacts with several anthocyanin-related bHLH transcription factors to further activate the promoters of key anthocyanin biosynthesis genes (Shen *et al.* 2014). However, whether and how *CYP707A* genes are involved in the regulation of ABA in cherry fruit ripening remains unclear.

In this study, four *CYP707A* enzymes were identified in sweet cherry. *PacCYP7072* was found to be the key gene regulating ABA levels during fruit ripening, while *PacCYP7071* regulates the response to dehydration stress during fruit development.

## Materials and methods

### Plant materials and treatments

Sweet cherry (*Prunus avium* L. cv. Satonishiki) fruits were collected from 10-year-old cherry trees grown in an experimental orchard at the China Agricultural University (Beijing, PR China) in the spring of 2013. Fruits were sampled at 21, 25, 29, 32, 36, 40, and 43 d after full bloom (DAFB). All fruits were collected from the middle of the branch and fresh fruits were used for the determination of pulp firmness, total soluble sugar (TSS), anthocyanin accumulation, and ABA content. Fresh fruits were frozen in liquid nitrogen immediately after separation and stored at –80 °C for RNA extraction.

### Fruit dehydration

In order to evaluate the effect of dehydration stress, 120 fruits were harvested at the de-greening stage 32 DAFB and divided evenly into two groups. The first group (control) was stored at 24 °C under high relative humidity (RH; 100%). The second group (dehydration stress) was stored at the same temperature and subjected to identical treatments but under low RH (45%; dehydrated fruits). Three days after treatment, the second group was transferred from 45% RH to 100% RH for a 1 d recovery period. ABA content and gene expression in the pulp were determined 0, 3, and 4 d after treatment. Each individual fruit was weighed immediately after harvest and weighed again before sampling to calculate the rate of water loss (the ratio of the decreased fruit weight to the initial fruit weight).

In order to evaluate the effect of dehydration stress on *PacCYP707A1/2*-RNAi-treated fruits, cherry fruits were harvested and divided into two groups 7 d after being treated with the *PacCYP707A1/2*-RNAi TRV vector. Each group contained 30 control fruits, 30 *PacCYP707A1*-RNAi-treated fruits, and 30 *PacCYP707A2*-RNAi-treated fruits. Group I fruits were stored in 100% RH as a control. Group II fruits were stored in 45% RH, and fruits were sampled 0, 1, 2, and 3 d after treatment. Fruits were immediately frozen in liquid nitrogen, powdered, mixed, and stored at –80 °C for further use. The rate of water loss was calculated as described above.

### Construction of the viral vector and agroinoculation

The pTRV1 and pTRV2 virus-induced gene-silencing vectors (Liu *et al.*, 2002) were kindly provided by YL Liu (School of Life Science, Tsinghua University, Beijing, China). A specific cDNA fragment of *CYP707A1* or *CYP707A2* gene was ampliﬁed using appropriate primers (Table 1), and the ampliﬁed fragments were cloned into *Eco*RI/*Sac*I-digested pTRV2. *Agrobacterium tumefaciens* strain GV3101 containing pTRV1, pTRV2, and pTRV2-*CYP707A1/2* were used for RNAi. Sixty fruits from three independent cherry trees grown in the experimental orchard were selected for inoculation and the *CYP707A1/2*-RNAi TRV vector was injected into each basal pedicel 28 DAFB (de-greening stage). Fruits were evaluated 7 d after treatment.

**Table 1.** The specific primers used for VIGS in this study

Gene name	
*PacCYP707A1* F-v, GU559987 (GenBank)	CGGGAATCTCTTCAGTCCTGGGAAGGCCGCTTGAT
*PacCYP707A1* R-v, GU559987 (GenBank)	CGGGAGCACCCTCCTCTTCTTCTTTTGTCCTC
*PacCYP707A2* F-v, GU559988 (GenBank)	CGGGAATCGTGGACAACATCATCGGGGTAAT
*PacCYP707A2* R-v , GU559988 (GenBank)	CGGGAGCGGAACAAAGGCAGTACCTTCCATCCCT

### Determination of fruit-soluble solids content

Ten randomly selected fruits per treatment were juiced to determine the soluble solids content (SSC) every four days from 21 DAFB. Data were obtained by squeezing the mesocarp with a Pal-1 pocket refractometer (ATAGO, Tokyo, Japan; units, ^o^Brix).

### Determination of fruit firmness

Cherry fruits were harvested at different ripening stages and the pulp firmness of 15 fruits was determined after removal of the skin on each side of the fruit suture using a KM-model fruit hardness tester (Fujihara Co., Japan). The units of pulp firmness used in this study were kg cm^–1^.

### Anthocyanin extraction and determination

The anthocyanin concentration was determined by extracting peel with 1% HCl methanol and measuring the absorbance at wavelengths of 530nm and 657nm. The formula *A*=*A*_530_–0.25*A*_657_ was used to compensate for the contribution of chlorophyll and its degradation products to the absorption at 530nm (Rabino and Mancinelli, 1986). Anthocyanin concentrations are relative, and *A*=0.01 was equal to one unit (U). All measurements were repeated five times with an equal quantity of peel.

### Quantitative real-time PCR analysis

Total RNA was isolated from cherry fruit samples using the hot borate method (Wan and Wilkins, 1994). Genomic DNA was eliminated using an RNase-free DNase I kit (Takara, China) according to the manufacturer’s recommendations. For each RNA sample, quality and quantity were assessed by agarose gel electrophoresis. cDNA was synthesized from total RNA using the PrimeScript RT reagent kit (Takara) according to the manufacturer’s recommendations. Primers used for real-time PCR are listed in Table 2 and were designed using Primer 5 software (http://www.premierbiosoft.com/). *Actin* was used as an internal control, and the stability of its expression was tested in preliminary studies as previously described (Ren *et al.*, 2011). All primer pairs were tested by PCR. The presence of a single product of the correct size for each gene was conﬁrmed by agarose gel electrophoresis and double-strand sequencing (Invitrogen). Ampliﬁed fragments were subcloned into the pMD18-T vector (Takara), and used to generate standard curves through serial dilution. Real-time PCR was performed using a Rotor-Gene 3000 system (Corbett Research, China) with SYBR Premix Ex Taq (Takara). Each 20 μl reaction contained 0.8 μl of primer mixer (containing 4 μM of each forward and reverse primer), 1.5 μl cDNA template, 10 μl SYBR Premix Ex Taq (2x) mixer, and 7.7 μl water. Reactions were performed under the following conditions: 95 °C for 30 s (one cycle), 95 °C for 15 s, 60 °C for 20 s, and 72 °C for 15 s (40 cycles). Relative fold changes in expression were calculated using the relative two standard curves method in the Rotor-Gene 6.1.81 software (Invitrogen).

**Table 2.** Primers used in qRT-PCR experiments

Gene	Forward primer	Reverse primer	GenBank
*PacNCED1*	CTCCAGAGTTCCGTATGGTTTTC	TAGCTTCCCACAGGTAATTGTCC	GQ913652
*PacCYP707A1*	GGAAGCAGGTGGAGGACCATAAG	GTTGTGTCACGAGCAGCGAAAAT	GU559987
*PacCYP707A2*	GAACAATCACCACCACAAAGAACTG	CTTGCCGAGACCGATTTATTGTATG	GU559988
*PacCYP707A3*	GAGAAGCAGTGGAAGACGTGGAGT	CAAATTTTTCGGGATGAGGGAAGA	GU559990
*PacCYP707A4*	CACATACCCCAAAAGCAAAGAACGT	CCCAGGAGTCTGAGCCAGACACA	GU559989
*PacCHS*	AGGGTGCTCGTGTTCTTGTTGTGTGC	CTGTCGGGAAGGATGGTTTG	GU938683.1
*PacCHI*	CCGTCAGTCAAACCACCAGGCTCAG	TCCAGCCCCCTCACCCCT	GAJZ01004967.1
*PacF3H*	TGTGGAGGCTTGTGAGGATTGGGG	GTAAATGGCTGGAGACGATG	GAJZ01002766.1
*PacANS*	GCCTTTTTCGATCTTCCCAT	CTTCTCCAGCCTCCCTTCTT	JF740094
*PacDFR*	CAAGCCAACAATAAATGGGGTGCTAG	GCAGAGGATGTAAACACCAG	HM543571.1
*PacUFGT*	GGTGTTTGATGTGCTGATGG	GCTGGTTGTAAAGTTGTGGGG	CP000264.1
*PacACO1*	GACAAATGGATTGATGTTCCACCG	ACAGCATCACTCCCCGGATTGTA	GU380294.1
*PacMYBA*	GGTGGTCATTGATTGCTGGA	GTGATGTTTGTGATGGCGTA	JN166079
*PacACT1*	CTCCTCTCAACCCTAAGGCTAACAG	CAGTTGTACGACCACTGGCATACAG	FJ560908

### Construction and transformation of the *PacMybA1* promoter::*GUS* vector

The *PacMYBA1* promoter sequence was inserted into *Pst*I/*Bam*HI-digested *pBI121* vector and fused upstream to the *GUS* gene to replace the *CaMV 35S* promoter. The resultant construct was transformed into cherry fruits (controls) at 32 DAFB using particle bombardment as described by Sun *et al.* (2011). *CYP707A1/2*-RNAi-treated fruits were harvested 7 d after inoculation and subjected to the same procedures as control fruits (Sun *et al.*, 2011). The pedicel detaching zone was used for particle bombardment, after which the target area was immediately covered with parafilm and incubated in a tissue-culture room for 24h at 25 °C. GUS assays were carried out according to the method of Jefferson (1987).

### Particle bombardment of cherry fruits

Tungsten particles were coated with plasmid as follows: 50 μl of prepared tungsten particle suspension in a 0.15ml centrifuge tube was supplemented with 5 μg of plasmid, 50 μl of 2.5M CaCl_2_ and 20 μl of 0.1M spermine in that order, mixed by vortexing for 3min, and incubated on ice for 5min. Following centrifugation at 8 000rpm for 1min, the supernatant was removed and the particles were resuspended in 150 μl of 70% ethanol and vortexed for another 3min. Following centrifugation as before, particles were resuspended in 150 μl of 100% ethanol, incubated on ice for 5min, centrifuged, and finally resuspended in 60 μl of 100% ethanol. Plasmid-coated tungsten particles were vortexed for 30 s and 10 μl was deposited onto a macroprojectile which was placed in a Petri dish filled with anhydrous CaCl_2_. Each macroprojectile was coated with 0.83 μg of plasmid. A Scientz GJ-1000 nitrogen-driven particle delivery system (Ningbo Scientz Biotechnology Co., Ltd, China) was used, and a partial vacuum of 71mm Hg was used for all bombardments.

### Histochemical staining of GUS activity

The GUS assay buffer contained three components: A=basic phosphate buffer (50mM sodium phosphate pH 7, 1mM K_3_Fe(CN)_6_, 1mM K_4_Fe(CN)_6_, 10mM Na_2_EDTA, 0.1% (v/v) Triton X-100); B=anhydrous methanol; C=20mM X-Gluc:5- bromo-4-chloro-3-indolyl-β-d glucuronide cyclohexylammonium salt in dimethyl formamide solvent. Components A, B, and C were used in a 40:10:1 ratio. Following incubation in the tissue culture room for 24h at 25 °C, the parafilm was removed from the target area and the pulp surface layer was cut into thin (2–3mm) slices, placed in a 5ml centrifuge tube containing 3ml assay buffer and incubated for 24h at 37 °C. To improve visual clarity, green or red pulp was discoloured with 70% ethanol.

## Results

### Expression of PacCYP707A genes during cherry fruit development and ripening

**Fig. 1.**
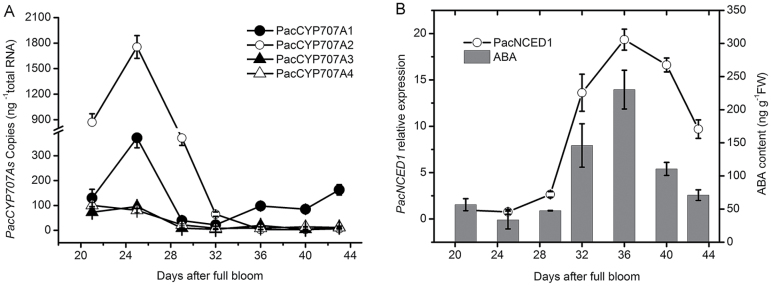
Changes in the expression of genes involved in ABA biosynthesis (*PacNCED1*), ABA catabolism (*PacCYP707As*), and ABA content during development and ripening of sweet cherry fruits. (A) Absolute transcript level profiles of four *PacCYP707As* during fruit development. (B) Relative transcript level profiles of *PacNCED1* and ABA accumulation during development. Expression was measured by qRT-PCR with *actin* as an internal control.

Sweet cherry fruits were sampled at seven visually distinct ripening stages based on fruit colouring and days after full bloom (DAFB) as follows: mid green (MG), 21 DAFB; big green (BG), 25 DAFB; de-greening (DG), 28 DAFB; yellow (YW), 32 DAFB; initial red (IR), 36 DAFB; full red (FR), 40 DAFB; dark red (DR), 44 DAFB. ABA levels and expression of *PacNCED1*, a key enzyme in ABA biosynthesis, began to increase in pulp at 25 DAFB, coinciding with termination of pit hardening and a sharp decline in fruit firmness (Fig. 1). Both parameters peaked at 36 DAFB before declining (Fig. 1B). In pulp, *PacCYP707A1* transcript levels were relatively low at 21 DAFB but increased dramatically to a maximum at 25 DAFB (Fig. 1A), at which point the ABA content reached its lowest level. After 25 DAFB, *PacCYP707A1* expression dramatically decreased up to 32 DAFB, when the ABA content began to increase. After 32 DAFB, *PacCYP707A1* transcript abundance began to increase slowly up to 44 DAFB (Fig. 1A). The expression pattern of *PacCYP707A2* was similar to *PacCYP707A1*, increasing steadily up to 32 DAFB before declining to its lowest level at 36 DAFB. *PacCYP707A2* transcript levels were higher than the other three *PacCYP707As* genes. Expression of *PacCYP707A3 and PacCYP707A4* remained relatively low throughout fruit development and ripening compared with *PacCYP707A1* and *PacCYP707A2* (Fig. 1A). These results indicated that *PacCYP707A2* is mainly involved in regulating ABA during the onset of ripening, whereas *PacCYP707A1* is more important for regulating ABA content during the later stages of fruit ripening.

### Expression of *PacCYP707A* genes in response to dehydration

**Fig. 2.**
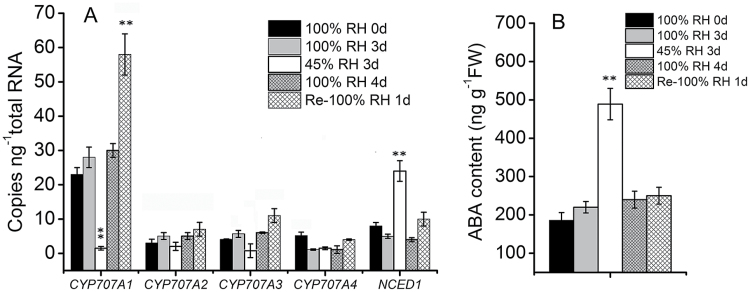
Effect of dehydration stress on ABA accumulation and expression of *PacNCED1* and four *PacCYP707As*. Cherry fruits were harvested at 32 d after full bloom and divided into three groups. Group 1 fruits (controls) were stored under 100% relative humidity (RH), group 2 (dehydration) were stored under 45% RH, and group 3 (recovery) were stored under 45% RH for 3 d then 100% RH for 1 d. All fruits were stored at or below 25 °C and sampled at 0, 3, and 4 d after treatment. Three biological replicates (*n*=3) were used for each analysis. (*^,^**) Values that are significantly different at the level of 0.05 and 0.01, respectively.

To investigate the regulatory roles of *PacCYP707A* genes, gene expression patterns in developing fruits in response to dehydration stress were analysed. Fruits harvested at 32 DAFB were divided into two groups and stored under either low RH (45%) or high RH (100%) conditions for 3 d. Fruits lost 16% of their water content in 45% RH, and ABA content and *PacNCED1* transcript levels were significantly elevated in dehydrated fruits compared with the controls (Fig. 2). By contrast, expression of *PacCYP707A1* was significantly down-regulated following dehydration, and *PacCYP707A3* expression was also down-regulated, albeit to a lesser extent, and expression of *PacCYP707A4* and *PacCYP707A2* was unaffected by dehydration (Fig. 2). *PacCYP707A1* underwent the most dramatic change in expression, suggesting this may be the primary drought-responsive member of the *PacCYP707A* gene family during fruit ripening.

### *PacCYP707A2* silencing promotes fruit colouring and ripening

**Fig. 3.**
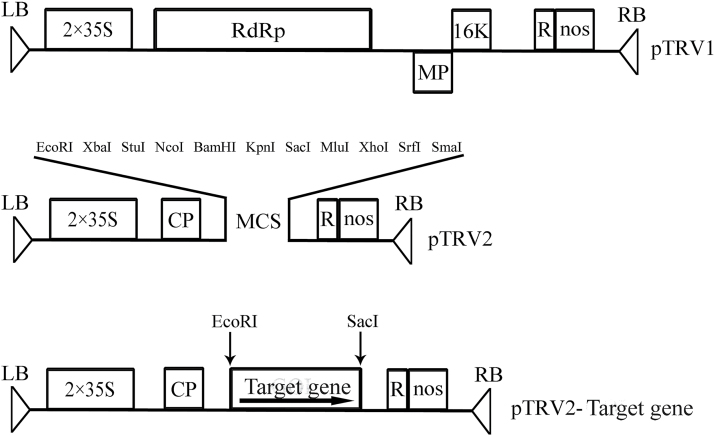
Construction of pTRV1, pTRV2, and pTRV2-derivative gene-silencing vectors as described by Liu *et al.* (2002). TRV cDNA clones were placed between duplicated CaMV 35S promoters and the nopaline synthase terminator in a T–DNA vector. pTRV2-target gene (sense orientation) was constructed to assess the ability of TRV vectors to suppress expression of the target gene in cherry fruits. RdRp, RNA-dependent RNA polymerase; 16K, 16kDa cysteine-rich protein; MP, movement protein; CP, coat protein; LB and RB, left and right borders of T–DNA, respectively; R, self-cleaving ribozyme; MCS, multiple cloning sites.

To clarify the role of *PacCYP707A2* in the regulation of ABA during fruit ripening further, ABA concentration and the expression of ABA-associated genes were measured in both RNAi-treated (Fig. 3) and control fruits. Injection of *PacCYP707A2*-RNAi TRV vector into growing fruits at 28 DAFB (de-greening stage) resulted in a faster accumulation of red colour (Fig. 7A) and more rapid ripening than controls 6 d after RNAi-treatment (Fig. 7F, control fruit on the right). At 12 d after RNAi-treatment (DAT), the time of fruit harvesting, pulp colour was more intensely red (Fig. 7E) than both non-silenced control fruits (Fig. 7C) and *PacCYP707A1*-RNAi-treated fruits (Fig. 7D). Indeed, treatment with *PacCYP707A1*-RNAi had no effect on pulp colour at 6 DAT (Fig. 7G, control fruit on the right), although pulp colour was slightly redder (Fig. 7D) than controls at the harvesting stage (Fig. 7C), but was much less intense than *PacCYP707A2*-RNAi-treated fruits. In *PacCYP707A1/2*-RNAi-treated fruits, the ABA content was higher than controls at 12 DAT (harvest stage) (Fig. 5).

### *PacCYP707A2* silencing affected the expression of ABA-responsive genes

**Fig. 4.**
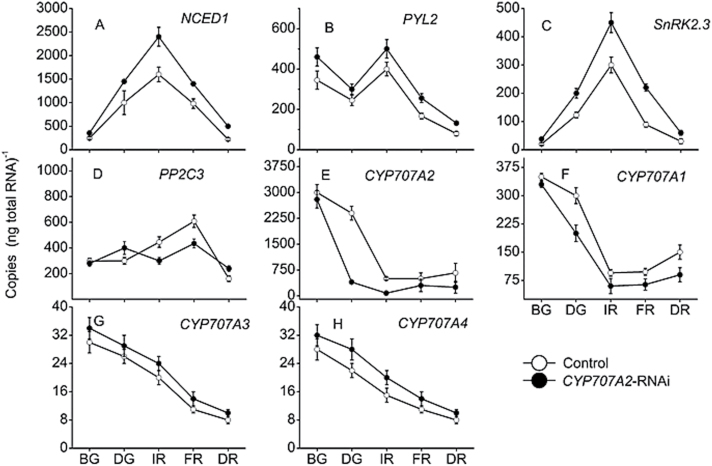
Expression of ABA-responsive genes in control and *PacCYP707A2*-RNAi-treated fruits during development and ripening. Growing fruits were injected with *PacCYP707A2*-RNAi TRV vectors at 28 d after full bloom (BG stage). Fruits were sampled at the big green (BG), de-greening (DG), initial red (IR), full red (FR), and dark red (DR) stages following injection. *Actin* was used as an internal control.

**Fig. 5.**
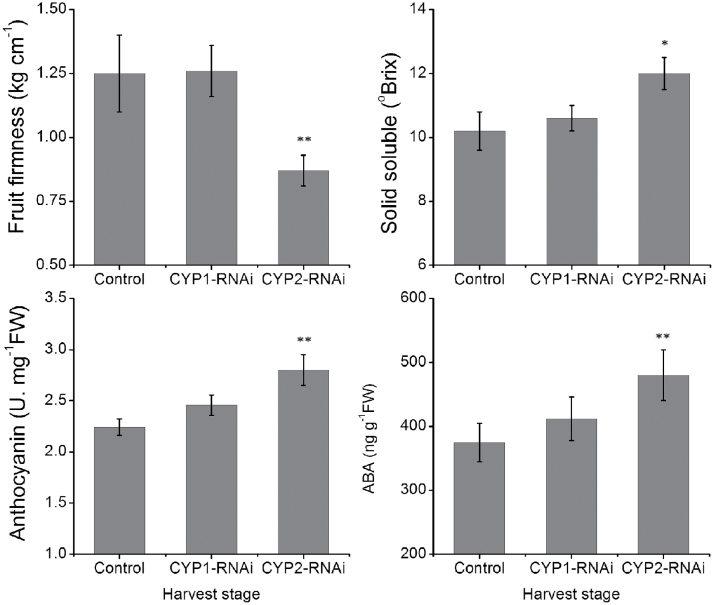
Changes in fruit firmness, solid soluble, anthocyanin, and ABA content in control, *PacCYP707A1*-RNAi-treated and *PacCYP707A2*-RNAi-treated fruits. A total of 10 fruits from each group were harvested at the full red stage. Three biological replicates (*n*=3) were used for each analysis. (*^,^**) Values that are significantly different at the level of 0.05 and 0.01, respectively.

**Fig. 6.**
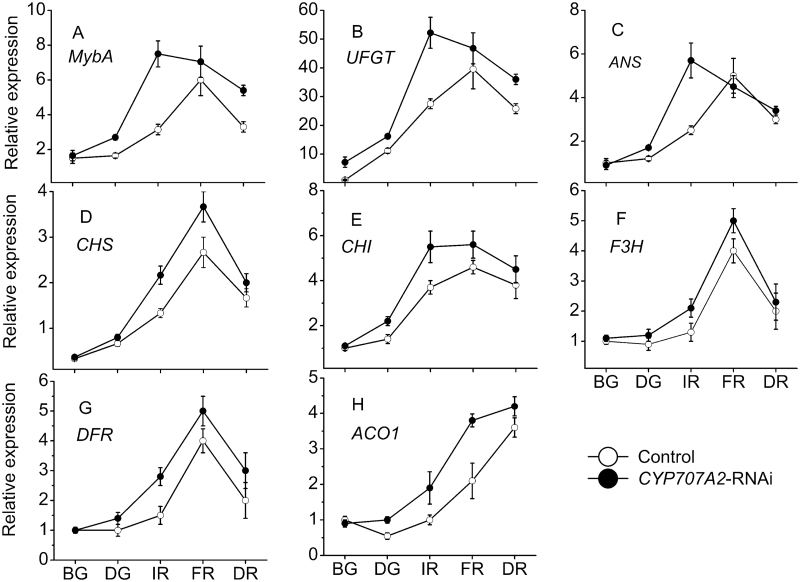
Changes in the relative expression of anthocyanin- and ethylene-associated genes and the *MybA* transcription factor in control and *PacCYP707A2*-RNAi-treated fruits during development and ripening. Fruits were injected with *PacCYP707A2*-RNAi TRV vectors at 28 d after full bloom and sampled at 0 (BG), 7 (DG), 9 (IR), 11 (FR), and 14 (DR) d after injection. *Actin* was used an internal control.

*PacCYP707A2* expression was down-regulated to 15% of controls in *PacCYP707A2*-RNAi-treated fruits during the de-greening stage, while *PacCYP707A1* expression was down-regulated to 60% and *PacCYP707A3/4* expression was slightly up-regulated during the de-greening and initial red stages (Fig. 4). Expression of *PacNCED1* was up-regulated in *PacCYP707A1/2*-RNAi-treated fruits (Fig. 4). Three ABA receptor genes (*PYLs*), six 2C protein phosphatases (*PP2Cs*), and six subfamily 2 SNF1-related kinases (*SnRK2*s) were previously cloned from cherry fruits (Wang *et al.*, 2015). Of these, *PacPYL2, PacPP2C3*, and *PacSnRK2.3* were strongly expressed in sweet cherry fruits during ripening, but expression of the other genes was very low. In *PacCYP707A1/2*-RNAi-treated fruits *PacPYL2* and *PacSnRK2.3* were up-regulated, while *PacPP2C3* was down-regulated (Fig. 4). Fruit firmness was lower than controls in *PacCYP707A2*-RNAi-treated fruits, but was comparable with controls in *PacCYP707A1*-silenced fruits. The soluble solid, anthocyanin and ABA content were all clearly elevated in *PacCYP707A2*-RNAi-treated fruits compared with controls, but only slightly up-regulated in *PacCYP707A1*-RNAi-treated fruits (Fig. 5). The expression of anthocyanin synthesis pathway genes *PacCHS, PacCHI, PacF3H, PacDFR, PacANS*, and *PacUFGT* was up-regulated by *PacCYP707A2* silencing during initial red and full red stages (Fig. 6B–G). *PacMybA* expression was particularly elevated in *PacCYP707A2*-RNAi-treated fruits (Fig. 6A). In addition, ABA accumulation and *PacNCED1* transcript levels were up-regulated in *PacCYP707A2*-RNAi-treated fruits, and this significant increase in NCED activity led to the up-regulation of *PacACO1* that encodes ACC oxidase (Fig. 6H).

### *PacMybA1* promoter responded to both *PacCYP707A1*-RNAi-treated and *PacCYP707A2*-RNAi-treated fruits

**Fig. 7.**
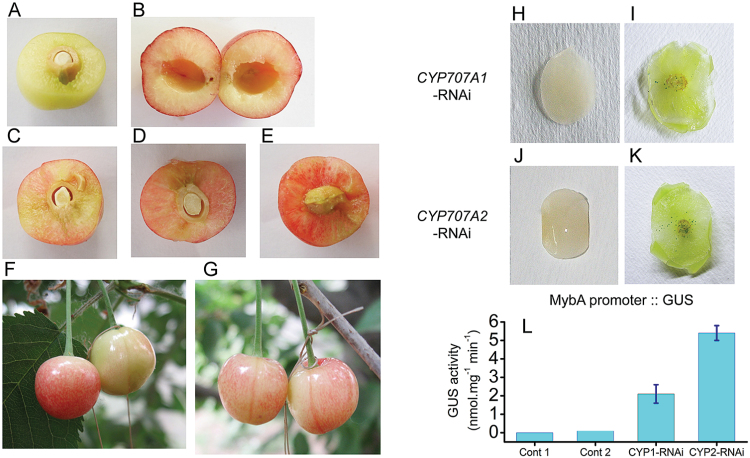
Phenotype of *PacCYP707A1/2*-RNAi-treated fruits. At 28 d after full bloom 90 fruits from three separate orchard-grown cherry trees were selected and divided into three groups of 30 (three replicates of 10 fruits) for virus-induced gene-silencing experiments. *PacCYP707A1*-RNAi, *PacCYP707A2*-RNAi, and control TRV vectors (no *CYP707A* gene) were injected into the pedicel of group 1, 2, and 3 fruits, respectively. Fruits were evaluated 7 d after inoculation. (A) Control fruits at 28 d after full bloom (DAFB); (B) control fruits at 43 DAFB; (C) TRV-vector-treated fruit (no *CYP707A* gene) at 43 DAFB; (D) *PacCYP707A1*-RNAi-treated fruit at 43 DAFB; (E) *PacCYP707A2*-RNAi-treated fruit; (F) *PacCYP707A2*-RNAi-treated fruit at 6 d after treatment (control fruit on the right); (G) *PacCYP707A1*-RNAi-treated fruit at 6 d after treatment (control fruit on the right). Panels H–L show histochemical GUS staining and GUS activity of (H) pulp from *PacCYP707A1*-RNAi-treated fruits; (I) pulp from *PacCYP707A1*-RNAi-treated fruits following bombardment with tungsten particles coated with *MybA* promoter::*GUS* vector; (J) pulp from *PacCYP707A2*-RNAi-treated fruit; (K) pulp from *PacCYP707A2*-RNAi-treated fruits following bombardment with tungsten particles coated with *MybA* promoter::*GUS* vector; (L) Comparison of GUS activity in the pulp of *PacCYP707A1*-RNAi- and *PacCYP707A2*-RNAi-treated fruits following bombardment with tungsten particles coated with MybA promoter::GUS vector and controls (Cont 1, PacCYP707A1-RNAi-treated fruit; Cont 2, PacCYP707A2-RNAi-treated fruits).

To investigate the effects of *PacCYP707A1/2*-RNAi in fruit colouring and ripening further, the responses of *PacMybA* promoter to *PacCYP707A1/2*-RNAi were detected. The promoter sequence was taken from the cherry fruits. It is found that the *PacMybA* promoter sequence contained two ABRE (ABA-responsive element) motifs with the core sequences TACGTG and CCTACGTGGC, respectively, but no ERE element (Shen *et al.*, 2014). To verify that *PacMybA1* exhibited a different response to *PacCYP707A1*-RNAi-treated and *PacCYP707A2*-RNAi-treated fruits, transient expressions of histochemical GUS staining and GUS activity were analysed in cherry fruits using a particle gun which expressed the constructions of *PacMybA1* promoter*::GUS* (Fig. 7H–L). For the transient expression of *PacMybA1* promoter*::GUS*, *PacCYP707A1*-RNAi (Fig. 7H, I) and *PacCYP707A2*-RNAi (Fig. 7J, K) treatments generated clear strong staining and high elevated GUS activity (Fig. 7L) compared with the control. The effect of *PacCYP707A2*-RNAi treatment was stronger than the *PacCYP707A1*-RNAi treatment (Fig. 7L).

### *PacCYP707A1* silencing improves tolerance to dehydration

**Fig. 8.**
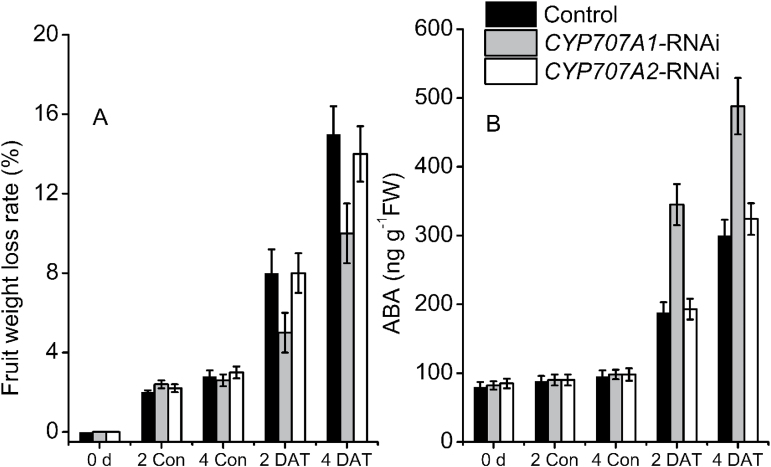
Rate of water loss and ABA content of *PacCYP707A1/2*-RNAi-treated fruits. Fruits were injected with *PacCYP707A1/2*-RNAi TRV vectors at 28 d after full bloom (DAFB). Fruits harvested at 40 DAFB were divided into two groups stored at 25 °C and 100% relative humidity (Group 1, control) or 25 °C and 45% relative humidity (Group 2, dehydration). Fruits were sampled at 0, 2, and 4 d after dehydration treatment. *Actin* was used as an internal control. Three biological replicates (*n*=3) were used for each analysis.

In *PacCYP707A1*-RNAi-treated fruits, expression of *PacCYP707A1* was down-regulated to 20%, while expression of *PacCYP707A3/4* was slightly up-regulated, and no obvious changes in *PacCYP707A2* expression were apparent at 7 DAT. Since *PacCYP707A1*-RNAi-treated fruits exhibited enhanced drought tolerance, the ABA content rate of water loss was examined during the initial red stage. Both control and *PacCYP707A1/2*-RNAi-treated fruits were harvested at 7 DAT and fruits were then incubated at 20 °C and 45% RH. The rate of water loss was lowest in *PacCYP707A1*-RNAi fruits, but was only slightly lower in *PacCYP707A2*-RNAi fruits than the controls at 2–4 d after dehydration (Fig. 8A). However, there were no large differences in the rate of water loss between the controls and *PacCYP707A2*-RNAi-treated fruits (Fig. 8A). *PacCYP707A1*-RNAi treatment therefore significantly improved resistance to dehydration stress compared with the controls. After 4 d of dehydration stress, control fruits were severely wilted, but this phenotype only appeared after a much longer dehydration period in *PacCYP707A1*-RNAi fruits. Indeed, almost all *PacCYP707A1*-RNAi fruits recovered within 4 d of rehydration, while only 70–80% of control or *PacCYP707A2*-RNAi-treated fruits survived this experimental regime. ABA levels were found to be higher in *PacCYP707A1*-RNAi-treated fruits than those in control and *PacCYP707A2*-RNAi-treated fruits (Fig. 8B). The ABA content in both control and *PacCYP707A1/2*-RNAi-treated fruits increased at 2–4 d after dehydration treatment, but ABA levels were highest in *PacCYP707A1*-RNAi-treated fruits (Fig. 8B). This may be due to the reduced expression of *PacCYP707A1* in these fruits. Together, these results suggest that *PacCYP707A1* plays a crucial role in tolerance to dehydration in cherry fruits.

## Discussion

In the fruits of the sweet cherry tree, endogenous ABA levels are determined by the dynamic balance of biosynthesis and catabolism (Fig. 1). Previous studies suggest *NCEDs* and *CYP707As*, respectively, mediate ABA biosynthesis and catabolism and their spatio-temporal expression is regulated at the transcriptional level (Saito *et al.*, 2004; Setha *et al.*, 2005). In many cases, expression of *NCED*s and *CYP707A*s is co-regulated by exogenous environmental stresses during plant development (Nitsch *et al.*, 2009). For example, ABA levels in tomato are primarily regulated by *LeNCED1* and *SlCYP707A2* (Sun *et al.*, 2012*a*; Ji *et al.*, 2014). Despite the fact that ABA catabolism plays an important role in regulating ABA levels in many physiological processes, evidence concerning the role of ABA catabolic genes in fruit development/ripening and tolerance to drought stress is scarce. Expression of *CYP707As* during fruit development/ripening and drought tolerance has been shown to exhibit an opposite trend to ABA content in a variety of fruits (Qin *et al.*, 1999; Ren *et al.*, 2011, Sun *et al.*, 2012*a*; Wang *et al.*, 2013). This trend was mirrored in sweet cherry fruits in the present study; expression of *PacCYP707A1/2* increased dramatically from 20–25 DAF but was low from 32–44 DAF, which was opposite to the ABA content (Fig. 1). These results indicate possible co-regulation of ABA content and *PacCYP707A1/2* expression at the transcriptional level.

*PacCYP707A2* may, therefore, play a primary role in regulating ABA levels during the initiation of cherry fruit ripening, and this was investigated using VIGS-induced *PacCYP707A2*-RNAi (Fig. 3). *PacCYP707A2* silencing resulted in increased ABA content at the fruit breaking and turning stages and the elevated ABA, in turn, stimulated the expression of the transcription factor *PacMybA* that regulates anthocyanin biosynthesis and accelerates fruit colouring and ripening (Figs 6, 7). *PacCYP707A2*-RNAi treatment significantly enriched anthocyanin accumulation in pulp at the fully ripe stage (Fig. 7E) compared with hte controls (Fig. 7B). This phenotype resembled that following suppression of *SlCYP707A2* genes in tomato, which also increased ABA levels and expedited fruit ripening (Ji *et al.*, 2014). Thus, *PacCYP707A2* may be a key gene in the regulation of ABA catabolism during the onset of ripening during cherry fruit de-greening. In addition, when *PacCYP707A1/2*-RNAi fruits were bombarded with tungsten projectiles coated with *PacMYBA* promoter::*GUS* plasmid DNA, both GUS staining (Fig. 7K) and GUS activity (Fig. 7L) were elevated compared with *PacCYP707A1*-RNAi fruits (Fig. 7I). This may be because ABA accumulation was higher in *PacCYP707A2*-RNAi fruits than in *PacCYP707A1*-RNAi fruits, and the *PacMYBA* promoter sequence contains two ABA response elements (ABREs) (Shen *et al.*, 2014). These results suggest that suppression of *PacCYP707A2* expression and over-expression of *PacNCED1* have a similar effect on the regulation of fruit ripening.

Water deficit in fruit is a direct result of osmotic stress caused by drought or excess salinity. It is well known that ABA content and ABA biosynthetic genes are up-regulated by abiotic stresses. In *Arabidopsis,* ABA is also involved in the reutilization and transport of Fe from roots to shoots under conditions of Fe deficiency (Lei *et al.*, 2014). In the present study, four *PacCYP707A* genes were differentially expressed in sweet cherry fruits in responsive to dehydration stress. *PacCYP707A1* expression decreased dramatically following dehydration but recovered after rehydration, and this trend was the opposite to the changes in ABA level (Fig. 2). *PacCYP707A1* may, therefore, be the major ABA catabolic gene responsible for negatively regulating ABA levels during dehydration in cherry fruits, which is consistent with previous reports (Saito *et al.*, 2004; Umezawa *et al.*, 2006). To investigate these findings further, VIGS experiments were performed, and *PacCYP707A1*-RNAi-treated fruits exhibited enhanced drought resistance and increased ABA accumulation relative to the controls (Fig. 8). This indicates that down-regulation of *PacCYP707A1* was responsible for the dehydration-induced ABA accumulation that led to improved drought tolerance. By contrast, silencing of *PacCYP707A2* did not prevent water loss, suggesting this is not a drought-associated gene in cherry fruits. In addition, changes in *PacNCED1* expression were less pronounced than those of *PacCYP707A1* under dehydration conditions, suggesting there may be *NCED* genes other than *PacNCED1* that respond to dehydration stress in this species.

In conclusion, of the four *PacCYP707A* genes identified in sweet cherry fruits, *PacCYP707A2* plays a crucial role in regulating ABA levels during fruit development and maturation, while *PacCYP707A1* is more involved in drought tolerance.

## Acknowledgements

We would like to thank Dr Yu-Le Liu (Qinghua University, China) for the pTRV vectors. We would like to thank the native English speaking scientists of Elixigen Company for editing our manuscript.
